# Is the Combination of Aerobic Exercise with Mat Pilates Better than Mat Pilates Training Alone on Autonomic Modulation Related to Functional Outcomes in Hypertensive Women? Secondary Analysis of a Randomized Controlled Trial

**DOI:** 10.3390/ijerph191710577

**Published:** 2022-08-25

**Authors:** Isabella da Silva Almeida, Letícia de Souza Andrade, Alessandra Martins Melo de Sousa, Gerson Cipriano Junior, Aparecida Maria Catai, Yomara Lima Mota, João Luiz Quagliotti Durigan

**Affiliations:** 1 Laboratory of Muscle and Tendon Plasticity, Graduate Program in Rehabilitation Science, Faculdade de Ceilândia, Universidade de Brasília, Distrito Federal, Brasília 72220-275, Brazil; 2 Physiotherapy School Clinic, Physiotherapy Department, Universidade Católica de Brasília, Distrito Federal, Brasília 71966-700, Brazil; 3 Laboratory of Exercise Physiology, Graduate Program in Rehabilitation Science, Faculdade de Ceilândia, Universidade de Brasília, Distrito Federal, Brasília 72220-275, Brazil; 4 Physiotherapy Department, Universidade Federal de São Carlos, São Paulo 13565-905, Brazil; 5 Health Education and Consultancy Department, PROCER Health Education and Consultancy, São Paulo 12916-398, Brazil

**Keywords:** cardiovascular disease, aerobic exercise, heart rate variability, Pilates method

## Abstract

Background: Although mat Pilates (MP) has become popular, the effects of MP in hypertensive women (HW) are not entirely clear. Here, we investigated the effects of 16 weeks of MP training contrasted with MP supplemented with aerobic exercise (MP+AE) and compared with a non-intervention group on autonomic modulation, cardiorespiratory fitness, strength, flexibility, performance of functional tasks, QOL, anthropometric variables, clinical BP, and heart rate. Methods: This is a three-arm, secondary analysis of an RCT. Sixty HW, aged 30 to 59 years, were allocated into: MP only (MP), MP+AE on a treadmill (MP+AE), and Control Group, without exercises. Assessments were performed before and after 16 weeks of training. Results: The ANOVA shows differences in between-group comparisons in the SDNN, rMSSD, and SD1 in the heart rate variability analysis, with increases in rMSSD, SDNN, and SD1 only in the MP, and this result was not found in the MP+AE group (*p* < 0.05). Differences were observed in the between-group comparisons in time in the cardiorespiratory exercise test (CPX), flexibility, and the waist-to-hip ratio, with changes in the MP+AE, differences in QOL, and increments in the MP and MP+AE (*p* < 0.05). Conclusions: MP increased the indices that reflect vagal and global cardiac autonomic modulation. MP+AE improved the CPX performance, flexibility, QOL, and anthropometric variables. These results suggest that MP supplemented or not with AE has promising effects in HW.

## 1. Introduction

Chronic noncommunicable diseases can be considered a global health problem. Among them, systemic arterial hypertension (SAH) stands out as being an important modifiable risk factor for cardiovascular diseases [1,2], in particular heart failure, peripheral vascular disease, and stroke [2,3,4]. One of the forms of non-drug treatment is physical exercise, which has been used to reduce blood pressure (BP) levels and has important clinical implications, contributing to improved general physical fitness [5,6]. Studies have shown that the practice of both aerobic (AE) and resistance exercises (RE) has benefits in this population [1,7], which supports the recommendation of AE supplemented for RE [6,8]. Currently, the Pilates method stands out as a type of RE [5] capable of promoting improvement in quality of life (QOL) [9], general functional capacity [10,11], and physical fitness [12]. The method is growing in popularity, especially among sedentary and middle-aged women who use it as their only health promotion modality [13].

The effects of the Pilates method have been little explored in the literature in hypertensive patients. We recently demonstrated that mat Pilates (MP) and MP with AE for 16 weeks of training reduced ambulatory BP in middle-aged women [14]. These results are in accordance with previous studies with MP in middle-aged [15] and hypertensive older women [16], justifying the use of the method in this population. In addition, in a study analyzing only one session, Rocha et al. [17] found a reduction in BP during the post-session recovery in hypertensive patients. Although previous studies have demonstrated that Pilates can decrease BP in hypertensive women [14,15,16], the mechanisms behind the process are poorly understood.

In a short-term follow-up, Rocha et al. [17] found, in addition to the reduction in BP, a decrease in parasympathetic markers assessed by the heart rate variability (HRV) during 60 min of recovery after a Pilates session. This fact did not support the theoretical premise which suggests that reductions in BP can be associated with changes in autonomic control due to a balance in autonomic modulation [18]. However, as far as we know, no studies have addressed the long-term changes in HRV to better understand the mechanisms of BP control in hypertensive patients. Furthermore, considering the popularity of the method and the recommendation of RE supplemented with AE for BP control, the effects of MP supplemented with AE (MP+AE) in hypertensive women are still little known. 

The purpose of this study was to investigate the effects of 16 weeks of MP training contrasted with MP+AE and compared with a non-intervention group (control group—CG) on autonomic modulation, cardiorespiratory fitness, strength, flexibility, performance of functional tasks (SFF), QOL, anthropometric variables, clinical BP, and heart rate (HR). We hypothesized that the participants of the intervention groups would exhibit an increase in indices that reflect parasympathetic modulation and an improvement in cardiorespiratory performance related to an improvement in the SFF analysis, QOL, anthropometric variables, and clinical BP and HR, especially in the MP+AE group.

## 2. Materials and Methods

This secondary analysis was embedded within a controlled, single-blind, randomized controlled trial (RCT) aiming to verify the long-term effects of MP and MP supplemented with AE training on ambulatory BP in hypertensive women [14]. Therefore, most of the methodology used in this secondary analysis was the same as for the RCT [14]. The study was carried out for 16 weeks at the School Clinic of the Catholic University of Brasília, conducted according to the Declaration of Helsinki. Approval for the project was obtained from the local ethics committee (CAAE: 99221818.9.0000.0029). The trial was registered on the clinical trial platform (https://clinicaltrials.gov/NCT03791307 accessed on 10 August 2022). Prior to participation, all participants were informed of the study’s objective, procedures, benefits, and potential risks, and signed the informed consent form. The study is reported according to the Consolidated Standards of Reporting Trials Statement for Randomized Trials of Nonpharmacologic Treatments and the Template for Intervention Description and Replication [19,20]. The details of the RCT have been reported previously [14], jointly with randomization, allocation concealment, and blinding [14]. In brief, the participants were randomized into three groups: MP: performed traditional MP exercises, MP+AE: performed traditional MP exercises with additional bouts of AE, and CG: the participants did not perform physical training during the experimental period. Outcome measures were assessed before the 16-week intervention period and 48 h after the final intervention session [14]. Clinical BP and HR, HRV, and QOL were evaluated on the first day of the assessment. The participants performed SFF tests, anthropometric analysis, and the cardiorespiratory exercise test (CPX) on a different day.

### 2.1. Subjects

The participants were recruited through advertising on social networks and flyers. The inclusion criteria were women, aged 30 to 59 years, sedentary or irregularly active for at least six months [21], clinically diagnosed with SAH [3], antihypertensive medication of any class, and medical permission to perform the exercises. The exclusion criteria were orthopedic problems that compromised the performance of the proposed exercises. Moreover, drug treatment changes, any cardiovascular events or symptoms that limited the participant from continuing the program, and frequencies below 75% in exercise sessions were considered as criteria to discontinue in the study [14].

For this secondary analysis, all 60 hypertensive women included in the previous RCT were randomized into 3 groups (age: 50.1 ± 6.2 years; body mass: 77.9 ± 17.7 kg; height: 159.0 ± 6.3 cm; BMI: 30.5 ± 6.2 kg/m^2^), and in this way, the baseline data are the same as for the previous study [14]. Four participants from the MP+AE and one participant from the CG did not complete the intervention and declined to attend the post-intervention assessments. The intention-to-treat analysis was applied, as described below [14]. Regarding HRV, 4 participants in the CG group, 8 participants in the MP group, and 7 participants in the MP+AE group were excluded from the analysis because they used beta-blockers, since this medication affects autonomic modulation. The HRV data of all participants are available in [14].

### 2.2. Training Protocol 

The sessions were administered for 16 weeks, twice a week. The sessions lasted 40 to 50 min, divided into warm-up and stretching, MP exercises, stretching, and cooling down phases [15]. MP sessions for both groups were intermittent, utilizing a work/rest ratio of 1/0.5 [22]. The sessions were performed following the design of three MP exercises (approximately 4 to 6 min) alternated with bouts of AE on a treadmill (MP+AE group) or periods performing a traditional MP exercise, named the shell stretch (MP group) (approximately 2 to 3 min) [23], as reported in the previous study [14].

The intensity of the MP exercises was the same for both groups according to the rate of perceived effort on the Borg scale, ranging from 11 to 13 during the 1st to 8th weeks and 13 to 15 during the 9th to 16th weeks. The intensity of the AE on the treadmill ranged from 80% to 85% of the second ventilatory threshold (VT2) during the 1st to 8th weeks and 85% to 95% of the VT2 during the 9th to 16th weeks, monitored using an HR monitor (Polar^®^, FT1 model, Kempele, Finland) [14].

### 2.3. Outcomes

#### 2.3.1. Cardiac Autonomic Modulation

To assess the R-R intervals (R-Ri), an HR monitor (Polar^®^, RS800cx model, Kempele, Finland) was used. The R-Ri was recorded for 30 min in the resting supine position (REST), 10 min during the active standing position (STAND), and 30 min in the recovery period in the resting supine position (REC). The sampling rate was 500 Hz. The analysis was performed by linear methods, in the time and frequency domains, and by using nonlinear methods. Data were recorded and downloaded for analysis using specific software (Polar Precision Performance, Polar). HRV indices were analyzed using Kubios HRV software (Biomedical Signal Analysis Group, Department of Applied Physics, Kuopio, Finland) [24]. For each participant, the 5 min of stable recording extracted from each analyzed experimental condition were considered. In the time domain, the following indices were obtained: mean R-Ri (R-Rmean), square root of the mean squared differences in successive R-Ri (rMSSD), a marker of cardiac vagal modulation, and standard deviation (SD) of normal R-Ri (SDNN), a marker of cardiac global modulation (sympathetic plus parasympathetic) expressed in milliseconds (ms) [25,26]. In the frequency domain, the bands corresponding to low-frequency spectral components (LF, ranging from 0.04 to 0.15 Hz, and representing sympathetic and vagal modulation, with sympathetic predominance) and high-frequency spectral components (HF, ranging from 0.15 to 0.4 Hz and representing vagal modulation) were obtained. These components were analyzed in normalized units (LFnu and HFnu) [25,26]. For HRV analysis by nonlinear methods, a quantitative analysis of the Poincaré plot was performed. This provides the SD of short-term variability of R-Ri (SD1) and the continuous long-term R-Ri variability (SD2) in ms. SD1 is considered a marker of cardiac parasympathetic modulation, while SD2 is considered a marker of both cardiac sympathetic and parasympathetic modulation [25,26].

#### 2.3.2. Cardiorespiratory Fitness 

The CPX was performed on a treadmill (Inbramed, Super ATL model, Porto Alegre, Brazil) with gas analysis (MGC Diagnostics^®^, Vo2000™ model, Saint Paul, MN, USA) to identify the first ventilatory anaerobic threshold (VT1), VT2, and cardiorespiratory fitness. Jointly, electrocardiographic analysis (Micromed, Wincardio model, Brasília, Brazil) using Ergo PC Elite software was performed. The VT1 and VT2 were identified by the ventilatory equivalent method [27]. The CPX was composed of increasing loads, with speeds of from 3.0 to 6.0 km/h and inclinations of 4% to 14%, without pauses between stages, until the participant’s exhaustion [28]. The following criteria were adopted as the maximum test: HR above 85% of the maximum predicted HR, respiratory exchange ratio > 1.10 [27], and the test was also interrupted at the discretion of the cardiologist responsible for the examination, as reported in the previous study [14].

#### 2.3.3. Strength, Flexibility, and Functional Tasks

Flexibility of the lower back and hamstring muscles was measured by the sit-and-reach test (cm) (Sanny^®^, São Bernardo do Campo, Brazil) [29]. In the dominant hand, handgrip strength (kilograms-force) was measured using a hand dynamometer (Jamar^®^, Exeter, UK). The tests of velocity to move from a sitting to a standing position (VST), from a supine to a standing position (VSP), and to put on sneakers and tie laces (VPS) were performed according to the protocol established by Raso [30]. For all tests, three attempts were performed, with an interval of one minute between them, and the best value was considered for each analysis, except for the VPS test, which was performed only once.

#### 2.3.4. Quality of Life

For the analysis of QOL, the World Health Organization Quality of Life/Brief (WHOQOL-brief) questionnaire was used, which enables self-assessment by the participant based on their perceptions. The WHOQOL-brief contains 26 questions that involve different aspects of everyday life and address 4 domains of QOL: physical, psychological, environmental, and social relations. The scores range from one to five for each component, representing from the worst condition to the best condition. The average score in each domain indicates the participant’s perception of their satisfaction in each aspect of their life related to their QOL [31].

#### 2.3.5. Anthropometry

Body weight (kg) was measured using a calibrated digital scale (Welmy, W300 model, São Paulo, Brazil), height (m) using a stadiometer (Sanny^®^, Brazil), and the body mass index (BMI) was calculated as the weight (kg) divided by height squared (m^2^). The waist (WC) and hip (HC) circumferences were measured using an anthropometric tape (Sanny^®^, Brazil) at the average distance between the last floating rib and the iliac crest and the largest perimeter of the gluteal region, respectively. The waist-to-hip (WHR) and waist-to-height (WHER) ratios were calculated using the following formulas: waist (cm)/hip (cm) and waist (cm)/height (cm), respectively.

#### 2.3.6. Clinical Blood Pressure and Heart Rate

The clinical BP evaluation was performed as proposed by the 7th Brazilian Guidelines on SAH [3], using an automatic arm BP monitor (Microlife^®^, BPA100 model, Widnau, Switzerland), and HR was monitored using an HR monitor (Polar^®^, FT1 model, Finland). Systolic blood pressure (SBP), diastolic blood pressure (DBP), mean blood pressure (MBP) (mmHg), and double product (DP) were measured three times with an interval of 1 min between measurements, after the participant had remained sitting at rest for 10 min. HR was monitored during rest, together with the clinical measurement of BP. The DP variable was estimated by multiplying SBP by HR (bpm × mmHg).

### 2.4. Statistical Analyses and Sample Size

The quantitative variables to characterize the sample are expressed as mean and SD or frequency distribution. The Levene’s test was used to evaluate the homogeneity of variances. Accordingly, parametric statistics were mainly performed. Two-way ANOVA was used to assess all outcomes with “time” (two levels: pre and post) and “groups” (three levels: MP, MP+AE, and CG) as factors. Post hoc analyses (Tukey’s HSD test) were performed when the threshold of significance was reached, and homoscedasticity was assumed. No Bonferroni correction was performed [32]. Effect sizes were determined using generalized eta squared (ηG2) for ANOVA [33]. Cohen’s d was used to determine the effect size. Cohen [34] provided benchmarks to define small (ηG2 > 0.01), medium (ηG2 > 0.06), and large (ηG2 > 0.14) effects. The statistical software used was STATISTICA (Version 12; StatSoft Inc., St Tulsa, OK, USA). In addition, SPSS statistical software (version 20; IBM Corp., Armonk, NY, USA) was utilized to obtain the confidence interval for within-group (follow-up minus baseline) and between-group differences at follow-up. All statistical tests were two-sided, and the significance level was set at *p* < 0.05. An intention-to-treat analysis was performed for all randomized participants. Missing data were replaced using the expectation-maximization method.

Since this study aimed to test and refine individual components of MP alone and MP+AE training in hypertensive women to assess the feasibility of a future larger trial, we did not undertake a formal sample size calculation. The total sample size of the main trial was used, and 20 participants were recruited per group [14]. However, for the HRV analysis, only participants who did not use beta-blockers were considered since this medication affects autonomic modulation.

## 3. Results

### 3.1. Cardiac Autonomic Modulation

**Figure 1:**
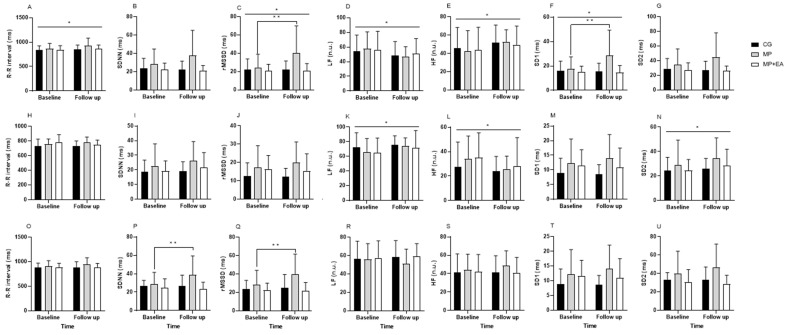
Comparisons for heart rate variability indices in the three moments analyzed, excluding participants who used beta-blockers (*n =* 41). (**A**–**G**) Supine moment at rest, (**H**–**N**) standing moment, and (**O**–**U**) recovery moment. CG: Control Group; MP: Mat Pilates Group; PM+AE: Mat Pilates Supplemented with Aerobic Exercise Group. R-R interval: the average of all normal R-R intervals; SDNN: standard deviation of R-R intervals; rMSSD: the square root of the mean squared differences in successive R-R intervals; LF: low-frequency band; HF: high-frequency band; SD1: variance of R-R intervals in a short time scale; SD2: variance of R-R intervals in a long time scale. * Indicates within-group effect (*p* < 0.05). ** Indicates MP group difference (*p* < 0.05).

Figure 1 and Table 1 show the data of HRV in the three periods analyzed. At REST, the within-group comparison revealed a statistical difference in the R-R interval (*p* = 0.01), rMSSD (*p* = 0.03), LFn.u. (*p* = 0.03), HFn.u. (*p* = 0.03), and SD1 (*p* = 0.04). The comparison between groups showed a statistically significant interaction in SDNN (*p* = 0.03), rMSSD (*p* = 0.01), and SD1 (*p* = 0.01). In addition, the MP group presented increased rMSSD (mean difference within-group = 15.7; *p* = 0.01) and SD1 (mean difference within-group = 11.1; *p* = 0.01). For the other groups, no differences were observed. 

During STAND, regarding within-group comparisons, a significant difference was observed in the LFn.u. (*p* = 0.04), HFn.u. (*p* = 0.04), and SD2 (*p* = 0.03). The comparison between groups did not show a statistically significant difference for any analyzed variables. In addition, during REC, no significant within-group difference was observed. However, the comparison between groups demonstrated a statistically significant difference in SDNN (*p* = 0.02) and rMSSD (*p* = 0.03). Furthermore, in the within-group analysis, the MP group showed an increase in SDNN (mean difference within-group = 10.07; *p* = 0.04) and rMSSD (mean difference within-group = 10.87; *p* = 0.04). For the other groups, no differences were observed. 

**Figure 2:**
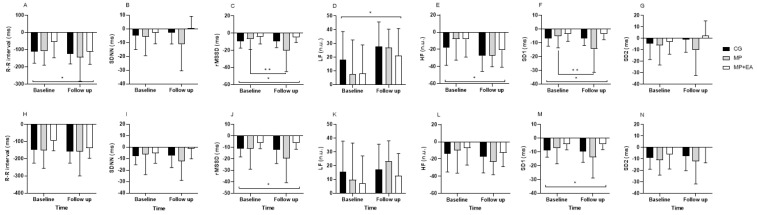
Comparisons for heart rate variability indices considering the delta of Stand and Supine and Stand and Recovery (Standing minus Recovery) for heart rate variability indices, excluding participants who used beta-blockers (N = 41). (**A**–**G**) Delta of Stand and Supine (Standing minus Supine), and (**H**–**N**) Stand and Recovery (Standing minus Recovery). CG: Control Group; MP: Mat Pilates Group; PM+AE: Mat Pilates Supplemented with Aerobic Exercise Group. R-R interval: the average of all normal R-R intervals; SDNN: standard deviation of R-R intervals; rMSSD: the square root of the mean squared differences in successive R-R intervals; LF: low-frequency band; HF: high-frequency band; SD1: variance of R-R intervals in a short time scale; SD2: variance of R-R intervals in a long time scale. * Indicates within-group effect (*p* < 0.05). ** Indicates MP group difference (*p* < 0.05).

Figure 2 and Table 2 show the data of HRV between-group and within-group comparisons considering the delta analysis. For the delta of STAND and REST (STAND minus REST), a significant difference was observed in the R-R interval (*p* = 0.02), rMSSD (*p* = 0.03), LFn.u. (*p* = 0.001), HFn.u. (*p* = 0.001), and SD1 (*p* = 0.03). The comparison between groups showed a statistically significant difference in rMSSD (*p* = 0.02) and SD1 (*p* = 0.02). Furthermore, the MP group showed changes in rMSSD (mean difference within-group = −13.3; *p* = 0.02) and SD1 (mean difference within-group = −9.3; *p* = 0.01). For the other groups, no differences were observed. In addition, a significant time effect was observed in the SD1 (*p* = 0.03) for the STAND and REC delta analysis (STAND minus REC). 

### 3.2. Cardiorespiratory Fitness

Regarding the within-group analysis for the cardiorespiratory fitness (Table 3 and Table 4), a significant difference in the relative VO2 at VT2 (*p* = 0.01) was observed. In addition, regarding the between-group analysis, a statistically significant difference was shown in the HR at VT2 (*p* = 0.03). At the max effort, we verified a significant time effect in the test time (*p* < 0.01), absolute power (*p* < 0.01), relative power (*p* < 0.01), and absolute VO2 (*p* = 0.02). Between-group comparisons showed a statistically significant effect in the test time (*p* = 0.02). Furthermore, an increase in test time in the MP+AE group was observed (mean difference within-group = 111.4; *p* < 0.01), while no differences were observed for the other groups.

### 3.3. Strength, Flexibility, and Functional Tasks

A significant within-group difference (*p* < 0.001) and between-group interaction (*p* = 0.002) was found in flexibility. In addition, an increase in flexibility in the MP+AE group was observed (mean difference within-group = 66.3; *p* = 0.005). For the other analyses, no differences were observed (Table 3 and Table 4).

### 3.4. Quality of life

A significant within-group effect was observed in the physical (*p* = 0.001), psychological (*p* < 0.001), social relationships (*p* = 0.02), and environment domains (*p* < 0.001), and overall QOL (*p* = 0.001). The comparison between groups showed a statistically significant difference in the psychological (*p* = 0.006) and environment domains (*p* = 0.03). In addition, the MP group (mean difference within-group = 1.3; *p* = 0.008) and the MP+AE group (mean difference within-group = 1.9; *p* = 0.002) showed an increase in the psychological domain, and the MP+AE group in the environment domain (mean difference within-group = 1.2; *p* = 0.01). For the other analyses, no differences were observed (Table 3 and Table 4).

### 3.5. Anthropometric Analysis

A significant within-group difference was observed in WC (*p* = 0.005), WHR (*p* = 0.02), and WHER (*p* = 0.003). In addition, the comparison between groups showed a statistically significant difference in the WHR (*p* = 0.01). Furthermore, the MP+AE group presented a decreased WHR (mean difference within-group = −0.038; *p* = 0.02). For the other analyses, no differences were observed (Table 3 and Table 4).

### 3.6. Clinical Hemodynamic Analyses

In the clinical hemodynamic analyses (Table 3 and Table 4), a significant within-group difference was observed in SBP (*p* = 0.03), MBP (*p* = 0.03), and DP (*p* = 0.01). However, the comparison between groups did not show a statistically significant interaction.

## 4. Discussion

The current study aimed to investigate the effects of 16 weeks of MP training contrasted with MP+AE compared to a non-intervention group on autonomic modulation, cardiorespiratory fitness, SFF, QOL, anthropometric variables, clinical BP, and HR. The main findings of this study support the assumptions that: (a) MP alone increases HRV indices that reflect vagal and global cardiac autonomic modulation, (b) MP+AE provided an increase in performance in the CPX, flexibility, QOL, and a reduction in the WHR, and (c) for both interventions, we found a reduction in clinical SBP, MBP, and DP.

Regarding the HRV during REST in the supine position, only in the post-MP training did our findings indicate an increase in cardiac vagal modulation, represented by an increase in rMSSD, and SD1 [18,26,35]. In addition, over the 16 weeks, we observed an increase in the R-R interval and a reduction in LFn.u. (that indicates sympathetic and parasympathetic modulation), which demonstrates better global autonomic modulation in this position [18,26]. These findings are essential since a decrease in HRV has been associated with an increased risk of coronary heart disease and cardiac mortality [36]. During STAND, we showed an increase in LFn.u. and a decrease in HFn.u., which may indicate better sympathovagal balance [26]; in addition, we also observed an increase in SD2, that represents the sympathetic and parasympathetic modulation [25,26]. During REC, we observed an increase in SDNN and in rMSSD for the MP group, showing better global autonomic modulation [18,26].

Another important point was that in this study, we analyzed HRV at three different moments. In fact, variations in HRV can be found in different postures, since the interactions of the systems are different [37,38], and there is a predominance of one system over the other depending on the position [38]. For example, in the supine position, there is a predominance of the parasympathetic nervous system [37,38]. We observed these alterations already reported in the literature [37,38,39] in our study and highlighted the fact that the MP group exhibited an increase in indices that reflect the vagal autonomic modulation or the global autonomic modulation over 16 weeks, especially in the supine position, which may indicate an improvement in autonomic modulation. The delta analysis reaffirms the findings in the three analyzed periods, highlighting an improvement in the indices that reflect the cardiac parasympathetic and global autonomic modulation.

The participants of the MP group performed only MP exercises instead of MP supplemented with AE, which may have contributed to increased HRV indices that reflect vagal and global cardiac autonomic modulation. In addition, other exercises such as yoga, which also focus on training the body and mind and have principles similar to MP, such as breathing, concentration, and slow movements, seem to increase HRV by increasing parasympathetic or reducing sympathetic modulation [40]. Thus, the characteristic of the method and its preservation throughout the session may be factors that contribute to long-term changes in HRV. On the other hand, this behavior did not seem to occur after an acute session. Rocha et al. [17] found, during the post-session recovery, reduced BP and parasympathetic response and an increase in SDNN and total power indices in hypertensive patients after a Pilates session (mat and apparatus), suggesting that the decrease found in BP post-session was not the result of increased parasympathetic modulation. In the present study, we found a reduction in clinical SBP. These data corroborate our previous RCT that found a reduction in ambulatory BP [14]. However, we did not verify the correlation between changes in BP and HRV in the present study. Thus, further analysis is needed to confirm our findings and the autonomic behavior in long- and short-term interventions addressing MP.

The data obtained through the CPX indicate an improvement in the performance of the test, even if there is no increase in VO2. In other words, the participants showed greater physical resistance when presenting max effort after a longer test time and, consequently, greater power, especially in the MP+AE group. Although MP is not considered a cardiorespiratory exercise, some studies have suggested that regular practice of the method can increase cardiorespiratory fitness in overweight people, heart failure patients, and healthy people [41], with an increase in VO2 in the ventilatory thresholds [42], peak VO2 [41], and maximum VO2 [42,43,44]. However, greater benefits in cardiorespiratory fitness seem to be achieved when the Pilates method is associated with another activity, such as running [12].

The evaluation did not reveal functional improvement in any SFF tests. However, it is essential to highlight the improvement in flexibility, especially in the MP+AE group. The improvement in flexibility has also been verified in previous studies due to its important participation in the performance of daily life movements [15,45,46,47]. In addition, regular practice in physical exercise programs improves flexibility and functional status and contributes to enhancing QOL [48]. Our findings showed an improvement in all domains of QOL analyzed, and this is particularly important for hypertensive patients since a better QOL can also motivate [49] and help hypertensive patients follow the recommendations for physical exercise [7,50].

Concerning anthropometric analysis, although no difference was observed in body mass and BMI, another interesting finding in this study was the significant reduction in WC, WHR, and WHER, suggesting changes in body composition. Previous studies addressing the Pilates method have also found results similar to the present study in hypertensive women [15,42,51]. These data are relevant since the increase and accumulation of adipose tissue in the abdomen are associated with an increased risk of cardiovascular and metabolic diseases, including high BP and stroke [52,53]. Thus, it is suggested that the Pilates method can promote beneficial effects, not only on the body composition, but can also have an impact on the reduction in cardiovascular and metabolic diseases.

One limitation of the present study is that we calculated the sample size based on secondary analysis using the sample from a previously published RCT [14]. The sample size calculation may have reduced the power of our outcome measures, indicating a possible type II error for these analyses. Moreover, it is important to highlight the small sample size for HRV analysis, where participants who used beta-blockers were excluded from the study. However, despite the small sample, we found positive results regarding HRV and the data on all participants are available [14]. Finally, further well-designed RCTs are needed to better understand the effects of MP and MP+AE in hypertensive women.

## 5. Conclusions

The results of the current study revealed that 16 weeks of training using MP alone increased indices that reflect cardiac vagal and global autonomic modulation. In addition, the MP+AE training had a greater effect in improving the performance of the CPX, flexibility, QOL, and anthropometric variables. Thus, these findings suggest that MP supplemented or not with AE has promising effects and may be an alternative to physical exercise for hypertensive women using antihypertensive medications.

## Figures and Tables

**Table 1:** Between-group and within-group comparisons for the heart rate variability indices in the three moments analyzed, excluding participants who used beta-blockers (N = 41).

	Within-Group				Between-Group			
	F	Power	Effect Size	*p-*Value	F	Power	Effect Size	*p-*Value
Data of heart rate variability indices								
Supine								
R-R interval (ms)	6.61	0.70	0.02	0.01 *	1.40	0.28	0.01	0.25
SDNN (ms)	1.77	0.25	0.007	0.19	3.65	0.63	0.03	0.03 *
rMSSD (ms)	4.54	0.54	0.03	0.03 *	4.79	0.76	0.06	0.01 *^†^
LF (n.u.)	4.80	0.57	0.03	0.03 *	0.23	0.08	0.003	0.78
HF (n.u.)	4.68	0.55	0.03	0.03 *	0.22	0.08	0.002	0.80
SD1 (ms)	4.40	0.53	0.02	0.04 *	4.86	0.76	0.06	0.01 *^†^
SD2 (ms)	0.95	0.15	0.003	0.33	2.96	0.54	0.02	0.06
Standing								
R-R interval (ms)	0.03	0.05	0.0002	0.86	1.50	0.30	0.02	0.23
SDNN (ms)	3.45	0.44	0.01	0.07	0.55	0.13	0.004	0.57
rMSSD (ms)	0.12	0.06	0.0006	0.72	0.66	0.15	0.006	0.52
LF (n.u.)	4.46	0.53	0.03	0.04 *	0.23	0.08	0.003	0.79
HF (n.u.)	4.48	0.54	0.03	0.04 *	0.23	0.08	0.003	0.79
SD1 (ms)	0.12	0.06	0.0006	0.73	0.65	0.15	0.006	0.52
SD2 (ms)	4.82	0.57	0.01	0.03 *	0.63	0.14	0.005	0.53
Recovery								
R-R interval (ms)	1.19	0.18	0.006	0.28	0.30	0.09	0.003	0.74
SDNN (ms)	2.96	0.38	0.01	0.09	4.04	0.68	0.04	0.02 *^†^
rMSSD (ms)	3.96	0.49	0.01	0.053	3.54	0.62	0.03	0.03 *^†^
LF (n.u.)	0.01	0.05	0.0002	0.88	0.35	0.10	0.007	0.70
HF (n.u.)	0.08	0.05	0.0009	0.76	0.25	0.08	0.005	0.77
SD1 (ms)	0.12	0.06	0.0006	0.73	0.65	0.15	0.006	0.52
SD2 (ms)	0.51	0.10	0.002	0.47	1.25	0.25	0.01	0.29

R-R interval: the average of all normal R-R intervals; SDNN: standard deviation of R-R intervals; rMSSD: the square root of the mean squared differences in successive R-R intervals; LF: low-frequency band; HF: high-frequency band; SD1: variance of R-R intervals in a short time scale; SD2: variance of R-R intervals in a long time scale. Effect size = generalized eta square (η*_G_*^2^). * *p* < 0.05. ^†^ MP group difference.

**Table 2:** Between-group and within-group comparisons considering the delta of Stand and Supine (Standing minus Supine) and Stand and Recovery (Standing minus Recovery) for heart rate variability indices, excluding participants who used beta-blockers (N = 41).

	Within-Group				Between-Group			
	F	Power	Effect Size	*p-*Value	F	Power	Effect Size	*p-*Value
Data of heart rate variability indices								
Delta Stand—Supine (Standing minus Supine)								
R-R interval (ms)	5.28	0.61	0.04	0.02 *	0.83	0.18	0.01	0.44
SDNN (ms)	0.00003	0.05	0.00003	0.99	2.22	0.42	0.01	0.12
rMSSD (ms)	4.86	0.57	0.03	0.03 *	4.04	0.68	0.06	0.02 *^†^
LF (n.u.)	12.2	0.92	0.11	0.001 *	0.53	0.13	0.01	0.58
HF (n.u.)	12.06	0.92	0.11	0.001 *	0.52	0.12	0.01	0.59
SD1 (ms)	4.78	0.56	0.03	0.03 *	4.17	0.70	0.06	0.02 *^†^
SD2 (ms)	0.44	0.09	0.003	0.50	1.36	0.27	0.01	0.26
Delta Stand—Recovery (Standing minus Recovery)								
R-R interval (ms)	1.62	0.23	0.01	0.21	0.67	0.15	0.009	0.51
SDNN (ms)	0.11	0.06	0.0005	0.73	3.15	0.57	0.02	0.054
rMSSD (ms)	4.14	0.50	0.01	0.04 *	2.43	0.46	0.02	0.10
LF (n.u.)	2.43	0.33	0.03	0.12	0.57	0.13	0.01	0.56
HF (n.u.)	2.92	0.38	0.03	0.09	0.44	0.11	0.01	0.64
SD1 (ms)	4.55	0.54	0.02	0.03 *	2.61	0.48	0.02	0.08
SD2 (ms)	1.06	0.17	0.006	0.30	0.99	0.21	0.01	0.37

R-R interval: the average of all normal R-R intervals; SDNN: standard deviation of R-R intervals; rMSSD: the square root of the mean squared differences in successive R-R intervals; LF: low-frequency band; HF: high-frequency band; SD1: variance of R-R intervals in a short time scale; SD2: variance of R-R intervals in a long time scale. Effect size = generalized eta square (η*_G_*^2^). * *p* < 0.05. ^†^ MP group difference.

**Table 3:** Mean (SD) of groups and mean (95% CI) within and between-group differences for the outcomes.

							Within-Group Difference (Follow-Up Minus Baseline)			Between-Group Difference at Follow-Up		
	CG (*n =* 20)		MP (*n =* 20)		MP+AE (*n =* 20)		CG (*n =* 20)	MP (*n =* 20)	MP+AE (*n =* 20)	CG vs. MP	MP vs. MP+AE	CG vs. MP+AE
	Baseline Mean (SD)	Follow-Up Mean (SD)	Baseline Mean (SD)	Follow-Up Mean (SD)	Baseline Mean (SD)	Follow-Up Mean (SD)	Mean Difference (95% CI)	Mean Difference (95% CI)	Mean Difference (95% CI)	Mean Difference (95% CI)	Mean Difference (95% CI)	Mean Difference (95% CI)
Data of cardiorespiratory fitness parameters												
HR at VT1 (bpm)	131.1 (13.9)	121.2 (12.0)	128.1 (21.2)	128.5 (17.1)	128.6 (16.2)	126 (13.9)	−9.9 (−17.6 to −2.0)	0.4 (−9.1 to 10.0)	−2.6 (−8.3 to 3.2)	−7.2 (−20.8 to 6.2)	2.4 (−11.4 to 16.4)	−4.8 (−12.9 to 3.3)
Relative VO_2_ at VT1 (mL/kg·min)	16.6 (2.3)	14.4 (2.3)	14.8 (2.9)	15.0 (2.9)	17.1 (3.8)	16.4 (3.1)	−2.1 (−3.3 to −1.0)	0.2 (−1.5 to 2.0)	−0.6 (−2.2 to 1.0)	−0.6 (−2.8 to 1.6)	−1.3 (−3.7 to 0.9)	−2.0 (−4.3 to 0.3)
HR at VT2 (bpm)	147.3 (14.8)	137.3 (14.2)	137.3 (23.0)	139.1 (17.2)	138 (16.7)	139.2 (12.5)	−10 (−17.5 to −2.4)	1.8 (−5.9 to 9.6)	1.2 (−5.1 to 7.5)	−1.7 (−11.8 to 8.2)	−0.1 (−13.3 to 13.2)	−1.8 (−12.6 to 8.9)
Relative VO_2_ at VT2 (mL/kg·min) *	19.5 (3.7)	17.2 (3.1)	18.3 (3.2)	17.7 (3.0)	19.2 (3.5)	19.0 (3.8)	−2.3 (−3.8 to −0.7)	−0.5 (−2.3 to 1.1)	−0.2 (−1.3 to 0.9)	−0.5 (−3.3 to 2.2)	−1.2 (−4.1 to 1.6)	−1.7 (−4.8 to 1.2)
Test time in max effort (ss) *	559.9 (123.8)	589.3 (120.6)	541.6 (117.7)	578.7 (126.2)	556.2 (112.6)	667.7 (131.4) ^†^	29.4 (−9.9 to 68.7)	37.1 (−15.6 to 89.8)	111.4 (64.2 to 15.7)	10.5 (−100.3 to 121.4)	−88.9 (−201.9 to 23.9)	−78.4 (−213.6 to 56.8)
HR in max effort (bpm)	164.6 (10.6)	158.5 (16.0)	158.6 (15.2)	154.9 (15.9)	156.0 (14.8)	157.1 (7.7)	−9.6 (−16.9 to −2.2)	−2.5 (−13.4 to 8.4)	−1.4 (−8.4 to 5.4)	−1.0 (−13.9 to 11.8)	−1.1 (−9.7 to 7.5)	−2.1 (−13.4 to 9.0)
Absolute power in max effort (W) *	265.3 (48.7)	274.7 (45.7)	247.4 (62.9)	262.1 (73.7)	237.4 (59.2)	275.1 (50.3)	9.4 (−7.9 to 26.8)	14.7 (−11.5 to 41.0)	37.6 (19.3 to 55.9)	12.5 (−35.9 to 61.1)	−12.9 (−43.2 to 17.3)	−0.3 (−37.8 to 37.2)
Relative power in max effort (W/kg) *	3.4 (0.7)	3.5 (0.7)	3.2 (0.7)	3.4 (0.9)	3.3 (0.6)	3.9 (0.7)	0.1 (−0.1 to 0.3)	0.1 (−0.1 to 0.5)	0.6 (0.3 to 0.9)	0.09 (−0.6 to 0.8)	−0.5 (−1.2 to 0.2)	−0.4 (−1.2 to 0.3)
Absolute VO_2_ in max effort (L/min) *	1.7 (0.3)	1.6 (0.3)	1.6 (0.2)	1.6 (0.2)	1.5 (0.3)	1.5 (0.2)	−0.1 (−0.2 to −0.02)	−0.01 (−0.1 to 0.9)	−0.02 (−1.1 to 0.05)	0.008 (−0.2 to 0.2)	0.07 (−0.08 to 0.2)	0.08 (−0.1 to 0.2)
Relative VO_2_ in max effort (mL/kg·min)	22.5 (3.9)	20.5 (3.8)	21.6 (3.4)	21.3 (3.4)	22.4 (3.7)	22.3 (4.1)	−1.9 (−3.3 to −0.5)	−0.3 (−2.0 to 1.3)	−0.1 (−1.3 to 1.1)	−0.7 (−3.5 to 2.0)	−1.0 (−4.0 to 2.0)	−1.7 (−5.3 to 1.8)
Strength, flexibility, and functional tasks												
Flexibility (cm) *	226.8 (82.3)	219.8 (82.8)	229.9 (75.5)	263.9 (66.1)	227.4 (103.4)	293.7 (51.3) ^†^	−7 (−20.4 to 6.2)	34 (11.2 to 56.8)	66.3 (22.2 to 110.4)	−44.1 (−99.1 to 10.8)	−29.8 (−73.7 to 14.1)	−73.9 (−135.3 to −12.5)
dominant hand strength (kgf)	28.6 (8.7)	27.3 (7.5)	29.1 (6.1)	29.7 (4.7)	27.6 (9.1)	27.8 (7.3)	1.1 (−4.1 to 6.3)	0.2 (−5.5 to 6.0)	−1.2 (−5.9 to 3.4)	2.1 (−4.3 to 8.5)	−0.2 (−2.4 to 1.9)	1.8 (−3.7 to 7.4)
VST (ss)	3.9 (1.2)	4.1 (1.9)	3.6 (1.0)	3.4 (1.0)	4.2 (1.3)	4.0 (2.6)	0.2 (−0.4 to 0.7)	−0.2 (−0.5 to 0.2)	−0.2 (−1 to 0.5)	0.6 (−0.7 to 2)	−0.6 (−2 to 0.9)	0.1 (−2 to 2.2)
VSP (ss)	4.8 (1.3)	5.3 (1.8)	4.3 (1.2)	4.3 (1.2)	5.5 (2.9)	5.5 (3.7)	0.5 (−0.2 to 1.3)	0 (−0.3 to 0.3)	0 (−1 to 0.9)	1 (−0.4 to 2.4)	−1.2 (−3.2 to 0.9)	−0.2 (−2.8 to 2.5)
VPS (ss)	29.1 (5.2)	30.9 (8.1)	28.2 (6.6)	28.8 (5.6)	31.2 (7.1)	29.3 (7.2)	1.8 (−2.1 to 5.9)	0.6 (−2.6 to 3.9)	−1.9 (−5 to 1.1)	2.1 (−3.9 to 8.2)	−0.4 (−4.4 to 3.5)	1.6 (−5.1 to 8.4)
Data of quality of life scores												
Physical *	12.2 (2.9)	12.3 (2.2)	12.8 (1.9)	14 (2.7)	12.3 (1.5)	14.2 (2.4)	0.1 (−0.9 to 1.1)	1.2 (−0.0 to 2.4)	1.9 (0.7 to 3.0)	−1.6 (−3.7 to 0.4)	−0.2 (−2.4 to 1.9)	−1.8 (−3.7 to 0.0)
Psychological *	13.4 (2.7)	13.6 (2.0)	13.3 (2.6)	14.6 (2.2) ^†^	12.3 (1.7)	14.2 (1.9) ^†^	0.2 (−0.5 to 0.9)	1.3 (0.4 to 2.2)	1.9 (1.2 to 2.5)	−1 (−2.6 to 0.5)	0.3 (−1.4 to 2.1)	−0.6 (−2.1 to 0.8)
Social relationships *	13.2 (3.2)	13.1 (2.7)	13.5 (2.8)	14.7 (2.0)	13.4 (2.2)	14.3 (1.7)	−0.1 (−1.1 to 1.0)	1.2 (−0.1 to 2.5)	0.9 (0.1 to 1.7)	−1.6 (−3.6 to 0.4)	0.4 (−1.3 to 2.1)	−1.2 (−2.9 to 0.5)
Environment *	12.4 (2.1)	12.3 (2.0)	11.6 (2.4)	12.4 (2.3)	11.1 (2.2)	12.3 (2.1) ^†^	−0.1 (−0.7 to 0.6)	0.8 (−0.0 to 1.7)	1.2 (0.7 to 1.7)	−0.1 (−1.3 to 1.1)	0.1 (−1.7 to 2.0)	0 (−1.9 to 2.0)
Overall QOL *	12.5 (3.4)	12.4 (2.3)	12 (2.1)	13.1 (2.5)	12.1 (2.3)	14.2 (2.5)	−0.1 (−1.6 to 1.4)	1.1 (−0.0 to 2.2)	2.1 (1.0 to 3.1)	−0.7 (−2.9 to 1.5)	−1.1 (−3.2 to 1.0)	−1.8 (−3.9 to 0.3)
Anthropometric data												
Body mass (kg)	79.9 (16.7)	78.7 (12.6)	78.1 (20.3)	75.8 (17.1)	74.3 (16.6)	70.3 (12.7)	−1.2 (−5.9 to 3.5)	−2.3 (−7.9 to 3.2)	−4 (−9.6 to 1.4)	2.9 (−10.5 to 16.4)	5.5 (−5.6 to 16.7)	8.4 (−3.4 to 20.3)
Height (cm)	158.6 (7.3)	158.4 (7.2)	160.9 (4.7)	161.1 (4.9)	157.4 (6.4)	157.9 (6.1)	−0.2 (−0.5 to 0.9)	0.2 (−0.3 to 0.7)	0.5 (0.0 to 0.8)	−2.7 (−7.6 to 2.2)	3.2 (−1.4 to 7.8)	0.5 (−4.9 to 5.9)
BMI (kg/m^2^)	31.6 (5.7)	31.2 (3.8)	30.0 (7.3)	29.1 (5.9)	29.9 (5.6)	28.2 (5.0)	−0.4 (−2.2 to 1.5)	−0.9 (−3.1 to 1.2)	−1.7 (−3.5 to 0.2)	2.1 (−2.4 to 6.8)	0.8 (−3.1 to 4.8)	3.0 (−1.3 to 7.4)
WC (cm) *	97.1 (7.0)	94.2 (8.1)	90.3 (12.2)	90.5 (11.9)	93.4 (12.8)	90.5 (11.2)	−2.9 (−4.9 to −0.7)	0.2 (−2.5 to 2.9)	−2.9 (−4.8 to −0.8)	3.7 (−5.3 to 12.7)	0 (−8.3 to 8.2)	3.6 (−5.5 to 12.8)
HC (cm)	111.0 (11.3)	110.7 (10.9)	109.6 (15.3)	108.3 (16.6)	103.9 (13.7)	105.3 (13.2)	−0.3 (−2.0 to 1.6)	−1.3 (−3.9 to 1.3)	1.4 (−0.9 to 3.7)	2.4 (−7.2 to 12.0)	3.0 (−9.8 to 15.9)	5.4 (−5.9 to 16.8)
Waist-to-hip ratio (cm) *	0.88 (0.07)	0.85 (0.08)	0.82 (0.07)	0.83 (0.07)	0.90 (0.07)	0.86 (0.07) ^†^	−0.03 (−0.04 to −0.001)	0.01 (−0.01 to 0.03)	−0.04 (−0.06 to −0.01)	0.02 (−0.04 to 0.07)	−0.03 (−0.08 to 0.03)	−0.01 (−0.07 to 0.05)
Waist-to-height ratio (cm) *	0.61 (0.05)	0.59 (0.05)	0.56 (0.07)	0.56 (0.07)	0.59 (0.07)	0.57 (0.06)	−0.02 (−0.03 to −0.004)	0 (−0.01 to 0.01)	−0.02 (−0.03 to −0.008)	0.03 (−0.02 to 0.09)	−0.01 (−0.06 to 0.03)	0.02 (−0.02 to 0.07)
Data of cardiorespiratory fitness parameters												
SBP (mmHg) *	118.5 (7.4)	118.4 (8.7)	117.7 (8.4)	115.6 (9.1)	122.2 (10.5)	114.7 (10.6)	−0.1 (−3.6 to 3.4)	−2.1 (−7.9 to 3.7)	−7.5 (−13.7 to −1)	2.8 (−5.7 to 11.3)	0.8 (−6.8 to 8.4)	3.6 (−3 to 10.2)
DBP (mmHg)	76.3 (10.5)	78.5 (9.2)	76.4 (6.9)	76.1 (7.7)	76.9 (9.1)	76.3 (7.4)	2.2 (−2.6 to 7)	−0.3 (−5.1 to 4.4)	−0.6 (−4.7 to 3.6)	2.4 (−5 to 9.8)	−0.2 (−7.1 to 6.6)	2.1 (−5.9 to 10.2)
MBP (mmHg) *	90.4 (7.6)	90.3 (7.0)	90.2 (6.6)	89.5 (5.4)	92.0 (8.4)	89.5 (7.8)	−0.1 (−1.2 to 1.1)	−0.7 (−2.6 to 1.2)	−2.5 (−4.5 to −0.3)	0.8 (−4.7 to 6.4)	0 (−6.0 to 5.8)	0.8 (−4.8 to 6.4)
HR (bpm)	70.9 (8.1)	70.6 (6.5)	71.2 (12.6)	67.1 (12.3)	68.7 (8.6)	67.1 (7.6)	−0.3 (−3.8 to 3.3)	−4.1 (−8.7 to 0.5)	−1.6 (−4.4 to 1.3)	3.5 (−5.1 to 12.2)	−0 (−8.7 to 8.6)	3.5 (−1.2 to 8.2)
DP (bpm X mmHg) *	8386.5 (893.1)	8358.6 (892.3)	8356.6 (1353.9)	7794.1 (1722.2)	8403.2 (1353.0)	7700.3 (1059.0)	−27.9 (−528.7 to 472.8)	−562.5 (−1274.5 to 149.5)	−702.9 (−1368.1 to −37.6)	564.5 (−645.9 to 1774.9)	93.7 (−1149.1 to 1336.7)	658.3 (−57.4 to 1374)

Footnote: SD: standard deviation; CI: confidence interval; CG: Control Group; MP: Mat Pilates Group; PM+AE: Mat Pilates Supplemented with Aerobic Exercise Group; VT1: first ventilatory threshold; VT2: second ventilatory threshold; VST: velocity of moving from sitting to standing position test; VSP: velocity of moving from supine to standing position; VPS: velocity to put on sneakers and tie the laces; SS: seconds; Overall QOL: Overall quality of life; BMI: body mass index; WC: waist circumference; HC: hip circumference; SBP: systolic blood pressure; DBP: diastolic blood pressure; MBP: mean blood pressure; HR: heart rate; DP: double product. Mean (standard deviation). Mean difference (confidence interval). * Within-group effect (*p* < 0.05). ^†^ Between-group interaction (*p* < 0.05).

**Table 4:** Between-group and within-group comparisons for the outcomes.

	Within-Group				Between-Group			
	F	Power	Effect Size	*p-*Value	F	Power	Effect Size	*p-*Value
Data of cardiorespiratory fitness parameters								
HR in VT1 (bpm)	3.366	0.43	0.01	0.07	1.993	0.39	0.01	0.14
Relative VO_2_ in VT1 (mL/kg·min)	3.773	0.47	0.02	0.057	2.700	0.51	0.02	0.07
HR in VT2 (bpm)	1.324	0.20	0.004	0.25	3.693	0.65	0.02	0.03 *
Relative VO_2_ in VT2 (mL/kg·min)	6.026	0.67	0.02	0.01 *	2.391	0.46	0.01	0.10
Test time in max effort (ss)	21.152	0.99	0.05	0.00002 *	4.116	0.70	0.02	0.02 *^,^**
HR in max effort (bpm)	1.45	0.21	0.01	0.23	0.79	0.17	0.01	0.46
Absolute power in max effort (W)	12.585	0.93	0.03	0.0007 *	2.217	0.43	0.01	0.11
Relative power in max effort (W/kg)	12.809	0.94	0.03	0.0007 *	3.150	0.58	0.02	0.05
Absolute VO_2_ in max effort (L/min)	4.810	0.57	0.01	0.03 *	2.068	0.40	0.008	0.13
Relative VO_2_ in max effort (mL/kg·min)	3.833	0.48	0.01	0.05	2.016	0.39	0.01	0.14
Strength, flexibility, and functional tasks								
Flexibility (cm)	14.4297	0.96	0.03	0.0003 *	6.7313	0.90	0.03	0.002 *^,^**
dominant hand strength (kgf)	0.0590	0.05	0.00008	0.80	1.0989	0.23	0.003	0.34
VST (ss)	0.1696	0.06	0.0004	0.68	0.4911	0.12	0.002	0.61
VSP (ss)	0.7023	0.13	0.001	0.40	0.7823	0.17	0.003	0.46
VPS (ss)	0.040	0.05	0.0002	0.84	1.350	0.27	0.01	0.26
Data of quality of life scores								
Physical	11.515	0.91	0.05	0.001 *	2.865	0.53	0.02	0.06
Psychological	28.863	0.99	0.06	<0.0001 *	5.583	0.83	0.02	0.006 *^,^^†,^**
Social relationships	5.102	0.60	0.01	0.02 *	1.598	0.32	0.01	0.21
Environment	11.612	0.91	0.02	0.001 *	3.411	0.61	0.01	0.03 *^,^**
Overall QOL	10.682	0.89	0.04	0.001 *	2.943	0.55	0.02	0.06
Anthropometric data								
Body mass (kg)	2.999	0.39	0.02	0.08	0.328	0.09	0.006	0.72
Height (cm)	1.08	0.17	0.0001	0.30	2.68	0.51	0.0005	0.07
BMI (kg/m^2^)	3.251	0.42	0.008	0.07	0.448	0.11	0.002	0.64
WC (cm)	8.510	0.81	0.007	0.005 *	2.554	0.49	0.004	0.08
HC (cm)	0.003	0.05	0.0	0.95	1.591	0.32	0.001	0.21
Waist-to-hip ratio (cm)	5.633	0.64	0.01	0.02 *	4.792	0.77	0.02	0.01 *^,^**
Waist-to-height ratio (cm)	9.203	0.84	0.008	0.003 *	2.480	0.47	0.004	0.09
Clinical blood pressure and heart rate								
SBP (mmHg)	4.61	0.56	0.03	0.03 *	2.14	0.42	0.02	0.12
DBP (mmHg)	0.106	0.06	0.0006	0.74	0.475	0.12	0.005	0.62
MBP (mmHg)	4.61	0.56	0.005	0.03 *	2.14	0.42	0.005	0.12
HR (bpm)	3.562	0.45	0.01	0.06	1.190	0.25	0.007	0.31
DP (bpm X mmHg)	6.104	0.68	0.03	0.01 *	1.389	0.28	0.01	0.25

VT1: first ventilatory threshold; VT2: second ventilatory threshold; VST: velocity of moving from sitting to standing position test; VSP: velocity of moving from supine to standing position; VPS: velocity to put on sneakers and tie the laces; SS: seconds; Overall QOL: Overall quality of life; BMI: body mass index; WC: waist circumference; HC: hip circumference; SBP: systolic blood pressure; DBP: diastolic blood pressure; MBP: mean blood pressure; HR: heart rate; DP: double product. Effect size = generalized eta square (η*_G_*^2^). * = *p* < 0.05. ** MP+AE group difference. ^†^ MP group difference.

## Data Availability

Not applicable.
